# ^18^F-FDG PET/CT findings in a patient with blastic plasmacytoid dendritic cell neoplasm and post-transplant lymphoproliferative disorder after hematopoietic stem cell transplantation: a case report

**DOI:** 10.3389/fmed.2023.1258310

**Published:** 2023-08-17

**Authors:** Jinzhi Chen, Xi Zhang, Linlin Ma, Yuan Gao, Zhanli Fu, Meng Liu

**Affiliations:** Department of Nuclear Medicine, Peking University First Hospital, Beijing, China

**Keywords:** blastic plasmacytoid dendritic cell neoplasm, hematopoietic stem cell transplantation, post-transplant lymphoproliferative disorder, ^18^F-FDG, PET/CT

## Abstract

**Background:**

Blastic plasmacytoid dendritic cell neoplasm (BPDCN) is an extremely rare hematopoietic malignancy, which originating from precursors of plasmacytoid dendritic cells. Allogeneic hematopoietic stem cell transplantation (HSCT) is normally considered in the treatment of BPDCN patients to acquire sustained remission. Post-transplant lymphoproliferative disorder (PTLD) is a group of conditions involving abnormal lymphoid cells proliferation in the context of extrinsic immunosuppression after solid organ transplantation (SOT) or HSCT. Herein, we report a patient with BPDCN, who suffered from PTLD after allogeneic HSCT.

**Case presentation:**

A 66-year-old man was diagnosed with BPDCN, confirmed by pathologic examination after splenectomy. The post-surgery ^18^F-fluoro-2-deoxy-D-glucose-positron emission tomography/computed tomography (^18^F-FDG PET/CT) showed multifocal ^18^F-FDG avidity in the left cheek, lymph nodes and bone marrow. The patient started chemotherapy, followed by allogeneic HSCT and immunosuppressive therapy. Four months after the HSCT, the patient developed intermittent fever and recurrent lymphadenopathy, accompanied with progressively elevated Epstein–Barr virus (EBV)-DNA both in serum and lymphocytes. ^18^F-FDG PET/CT was performed again and found multiple new enlarged ^18^F-FDG-avid lymph nodes, while the previous hypermetabolic lesions all disappeared. The pathology of mesenteric lymph node indicated a monomorphic PTLD (diffuse large B-cell lymphoma). Then the immunosuppressive medications were stopped and two cycles of Rituximab were given, and the follow-up CT scan indicated a complete response.

**Conclusion:**

When patients with BPDCN recurred new enlarged lymph nodes after allogeneic HSCT and immunosuppressive therapy, PTLD should be taken into consideration. ^18^F-FDG PET/CT may provide additional evidence for supporting or refuting the suspicion of PTLD, and suggest lesions accessible for biopsy.

## 1. Introduction

Blastic plasmacytoid dendritic cell neoplasm (BPDCN), originating from the precursor of plasmacytoid dendritic cells, is an extremely rare and aggressive hematopoietic malignancy (1). It typically involves skin, lymph nodes and bone marrow, while its exact incidence remains unknown (2). The diagnosis of BPDCN is mainly based on histology, immunohistochemistry, and flow cytometry (3, 4). ^18^F-fluoro-2-deoxy-D-glucose-positron emission tomography/computed tomography (^18^F-FDG PET/CT) is helpful in staging of BPDCN (5). Given the highly aggressive clinical behavior, the overall prognosis of BPDCN is poor (6–8). There have been no standard treatments for BPDCN, and leukemia-type chemotherapy followed by allogeneic hematopoietic stem cell transplantation (HSCT) can be considered as a consolidative strategy (9). Post-transplant lymphoproliferative disorder (PTLD) is a group of conditions involving abnormal lymphoid cells proliferation in the context of extrinsic immunosuppression after solid organ transplantation (SOT) or HSCT (10). Herein, we report a patient with BPDCN, who suffered from PTLD after allogeneic HSCT. The unique ^18^F-FDG PET/CT patterns at the initial diagnosis of BPDCN and at the time of PTLD after HSCT indicated that ^18^F-FDG PET/CT might be helpful for the differential diagnosis and suggesting lesions accessible for biopsy.

## 2. Case report

**Figure 1:**
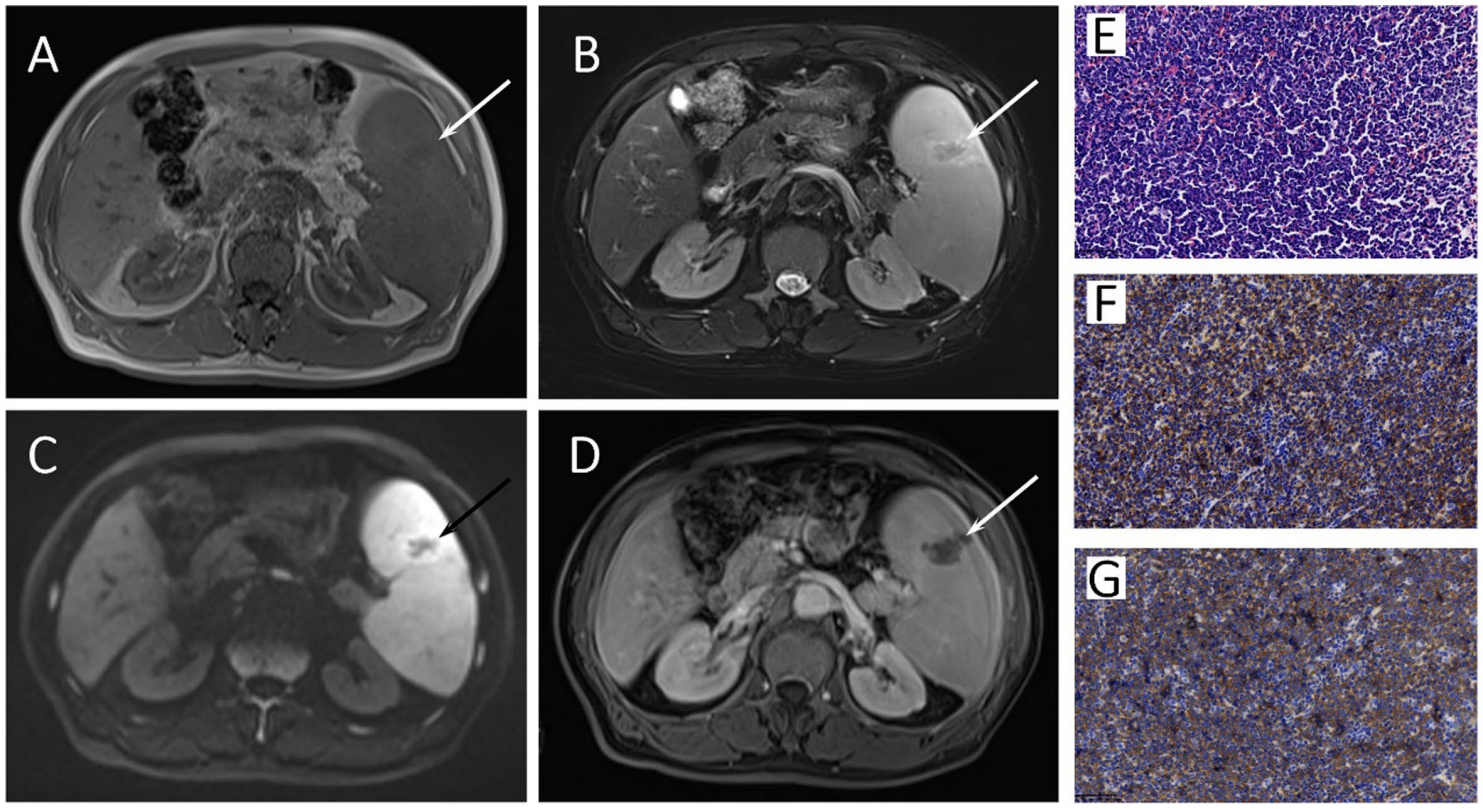
The axial image of T1-weighted **(A)**, Fat-suppressed T2-weighted **(B)**, diffusion-weighted **(C)**, and gadolinium-enhanced T1-weighted **(D)** MRI images showed massive splenomegaly with an abnormal signal lesion (arrows) in the spleen. The HE staining specimen of spleen **(E)** showed diffuse distribution of small to medium monomorphic round cells with moderate cytoplasm and inconspicuous nucleoli, and the neoplastic cells showed both positive for CD56 **(F)** and CD123 **(G)** in immunohistochemistry.

A 66-year-old man presented with continuous fever with the highest temperature of 38.6°C and left upper abdominal pain for 1 month. The laboratory examination showed a progressive decrease of blood platelets, with a minimum of 32 × 10^9^/L. Ultrasound examination showed splenomegaly and multiple enlarged lymph nodes in bilateral supraclavicular regions, bilateral axillas, and bilateral groins. Contrast-enhanced magnetic resonance imaging (MRI) revealed massive splenomegaly with an abnormal signal lesion in the spleen (Figures 1A–D), and multiple enlarged lymph nodes in the hepatic hilum and around the pancreas. Combining the patient’s progressive thrombocytopenia and massive splenomegaly, hypersplenism was considered, and splenectomy was performed subsequently. Hematoxylin and eosin (HE)-stained specimens of the splenic lesion revealed diffuse distribution of small to medium monomorphic round cells with moderate cytoplasm and inconspicuous nucleoli, and the result of immunohistochemistry (IHC) showed CD56 (++), CD123 (+), CD4 (++), CD43 (++), which was consistent with BPDCN involving the spleen (Figures 1E–G).

**Figure 2:**
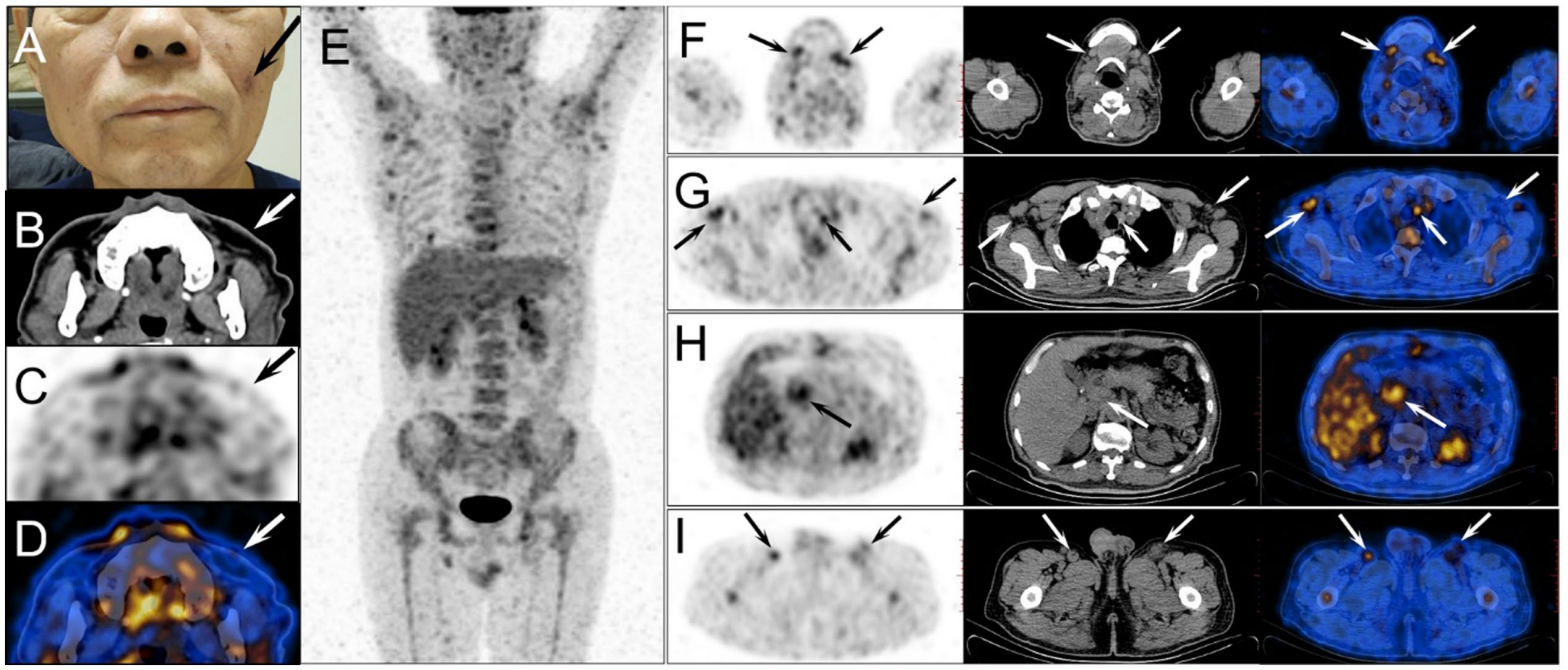
The physical examination revealed a brown rash on the patient’s left cheek [**(A)**, arrow]. The axial image of head CT revealed slightly skin thickening in the left cheek [**(B)**, arrow], with mild ^18^F-FDG avidity (SUVmax 1.1) on the corresponding PET [**(C)**, arrow] and PET/CT fusion [**(D)**, arrow] images. The body MIP image **(E)** demonstrated diffuse ^18^F-FDG uptake in bone marrow (SUVmax 4.0) and multiple metabolic foci as described in the following axial images. On the axial images of PET, CT and PET/CT fusion, multiple enlarged lymph nodes were found in bilateral submaxillary regions **(F)**, bilateral axillas and mediastinum **(G)**, hepatic hilum **(H)**, and bilateral groins **(I)** (arrows), which exhibited varying ^18^F-FDG avidity with the maximal SUVmax of 4.2 in the left submaxillary lymph nodes.

Further physical examination revealed a brown rash on the patient’s left cheek (Figure 2A), and ^18^F-FDG PET/CT was performed for evaluating the systemic involvement of BPDCN. The patient fasted for at least 6 h before PET/CT examination, and his blood sugar concentration on the day of examination was 6.2 mmol/L. PET/CT (Philips Gemini GXL, Philips Medical Systems, United States) scan was performed 60 min after the intravenous administration of ^18^F-FDG (3.7 MBq/kg body weight, provided by Atom high-tech Co., Ltd., Beijing, China). Image acquisition began with CT for attenuation correction and anatomical reference, using a standardized protocol with a tube voltage of 120 kV, a tube current of 100 mA/s, and a matrix of 512 × 512. Then PET scan was performed with a matrix of 144 × 144, 1.5 min per bed position, 8 bed positions. Pulmonary breath-holding CT was carried out with a tube voltage of 120 kV, a tube current of 100 mA/s. All images were reconstructed by Fusion Viewer software in the Extended Brilliance Workstation (EBW, Philips Medical Systems, United States).

The images revealed slightly skin thickening in the left cheek with mild ^18^F-FDG avidity with maximum standardized uptake value (SUVmax) of 1.1 (Figures 2B–D), diffuse uptake in the bone marrow with SUVmax of 4.0, and multiple metabolic enlarged lymph nodes with SUVmax of 4.2 (Figures 2E–I). The SUVmax of liver and blood pool was 3.3 and 2.1, respectively. The pathological findings of the left cheek rash, bone marrow, and cervical lymph node were all consistent with BPDCN.

Subsequently, the patient received chemotherapy with one cycle of VDLP (vincristine, daunorubicin, L-asparaginase and prednisone) and three cycles of CHOPE (cyclophosphamide, doxorubicin, vincristine, prednisone, and etoposide). Follow-up ultrasound showed that the previously enlarged lymph nodes disappeared, and bone marrow biopsy showed no abnormal cells. For maintaining a sustained remission, the patient underwent allogeneic HSCT and immunosuppressive therapy.

**Figure 3:**
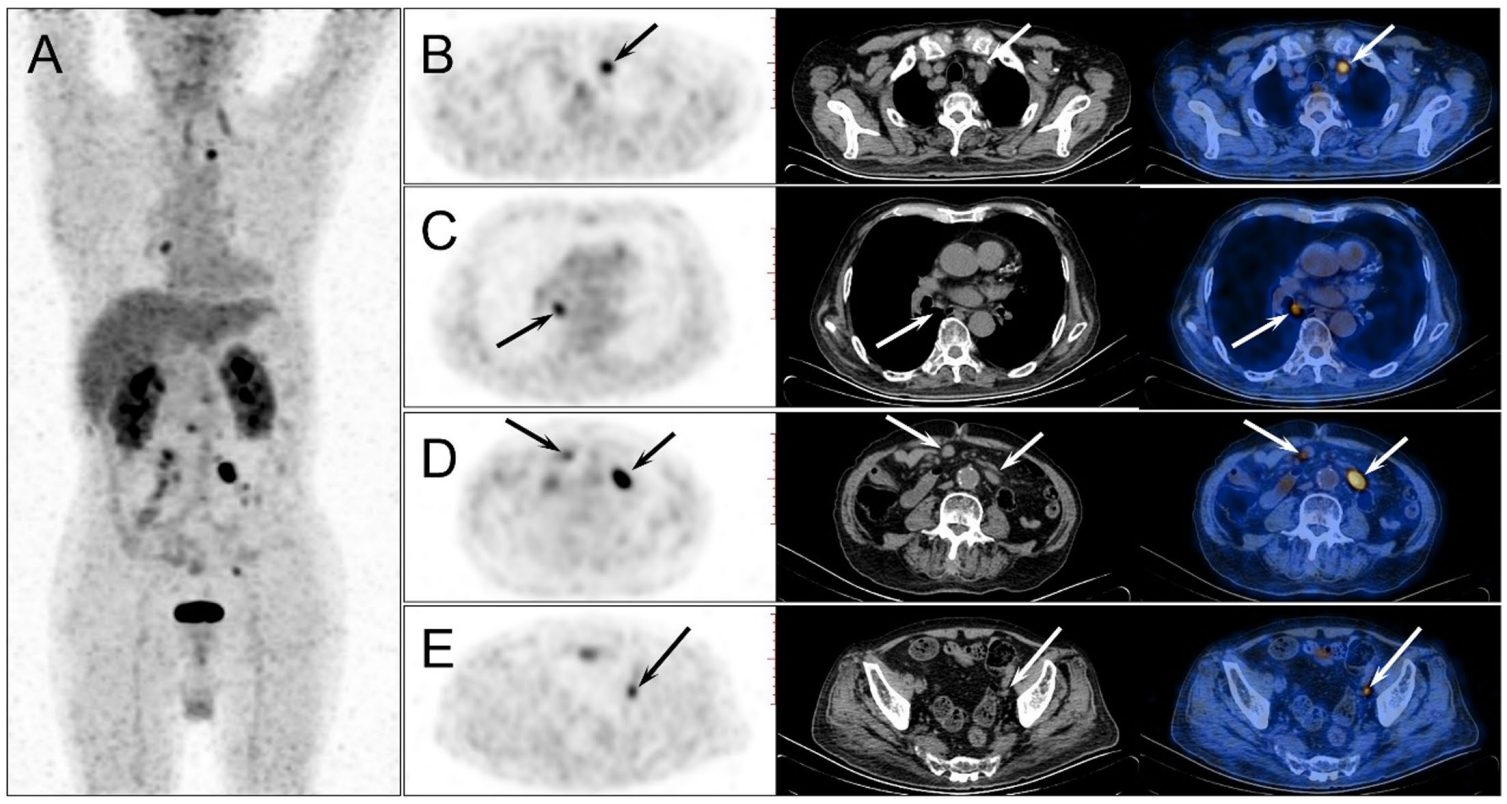
The second ^18^F-FDG PET/CT was performed 4 months after the HSCT. The MIP image **(A)** showed multiple new ^18^F-FDG-avid lesions in the chest and abdomen, while the previous hypermetabolic lesions all disappeared. On the axial images of PET, CT and PET/CT fusion, enlarged lymph nodes were observed in mediastinum **(B,C)**, and abdominal and pelvic cavity **(D,E)** (arrows), which demonstrated intense ^18^F-FDG uptake with the highest SUVmax of 13.9 in the mesenteric lymph node.

Four months after HSCT, the patient developed intermittent fever with the highest temperature of 39.0°C. The EBV-DNA both in serum and lymphocytes was gradually increased. All these clues leaded the clinicians to the suspicion of PTLD, but more evidences were required to exclude the acute infection, graft-versus-host disease (GVHD), graft rejection or malignancy relapse. Hence, the patient was referred to a second ^18^F-FDG PET/CT examination. On the examination day, the patient’s fasting blood sugar concentration was 7.5 mmol/L. The images showed multiple new ^18^F-FDG-avid enlarged lymph nodes in the chest and abdomen with SUVmax of 13.9, while the previous hypermetabolic lesions all disappeared (Figure 3). The SUVmax of liver and blood pool was 4.1 and 2.7, respectively.

**Figure 4:**
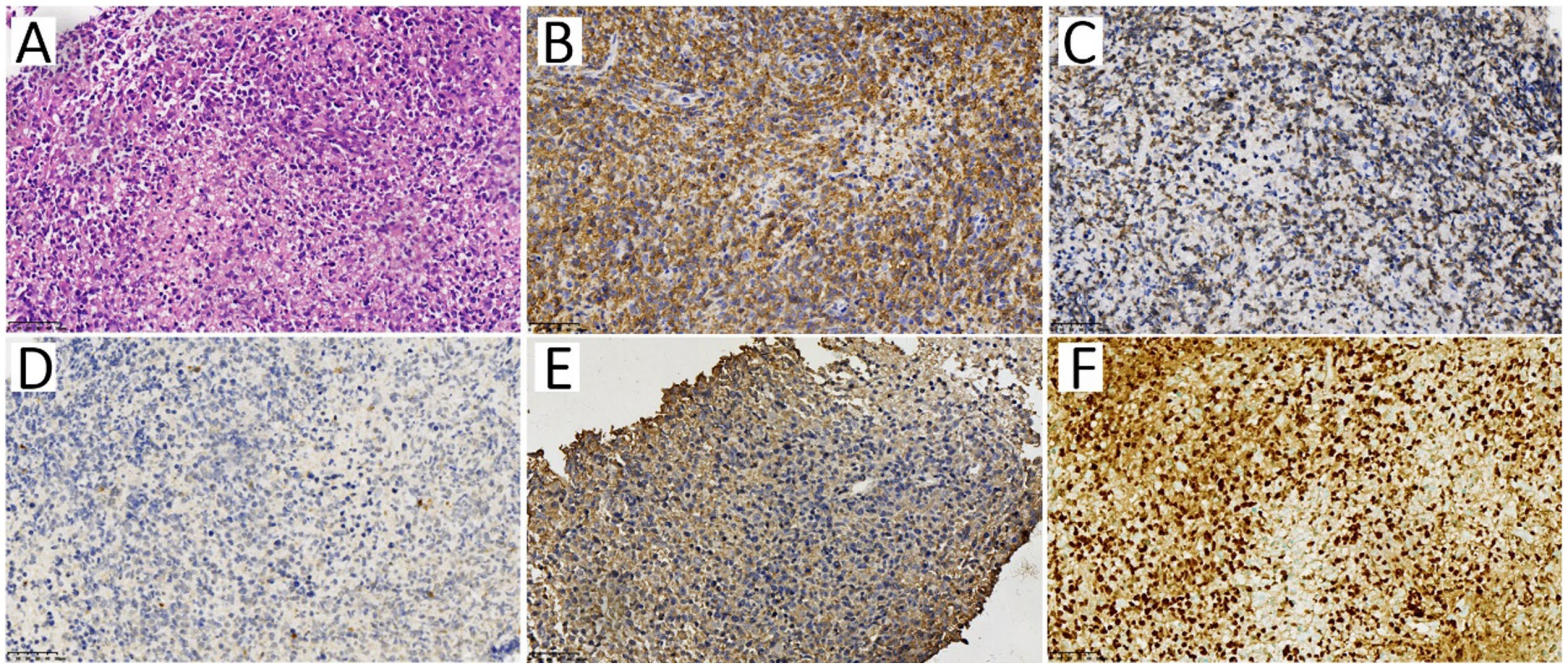
The pathology of the mesenteric lymph node. The HE staining specimen **(A)** showed diffuse medium-large cell infiltration with nuclear irregularity. Immunophenotype of the neoplastic cells showed CD20 **(B)** and Bcl-2 **(C)** were positive, while CD56 **(D)** and CD123 **(E)** were negative. The *in-situ* hybridization (ISH) staining of EBV-encoded RNA [EBER, **(F)**] showed EBV positive.

Based on the ^18^F-FDG PET/CT results, a core needle biopsy was performed on the ^18^F-FDG-avid mesenteric lymph node. The HE result showed diffuse medium-large cell infiltration with nuclear irregularity, and the IHC results showed CD20 (+++), CD3 (−), CD56 (−), CD10 (−), Bcl-6 (−), MUM1 (+), Bcl-2 (80%+), CD123 (−), with EBV-positive in the *in-situ* hybridization for EBV-encoded RNA (Figure 4). The pathology of the mesenteric lymph node proved a diffuse large B-cell lymphoma, indicating the diagnosis of monomorphic PTLD.

Then the immunosuppressive medications were stopped and two cycles of Rituximab were given. One month after the treatment, the EBV DNA in the patient’s serum and lymphocytes was undetectable. The follow-up CT showed the lesions disappeared completely, characterized as complete response.

## 3. Discussion

BPDCN, derived from precursors of plasmacytoid dendritic cells, is an extremely rare hematopoietic malignancy (11). It commonly affects elderly males, with a male/female incidence ratio of at least 3:1 (12, 13). Ninety percent of patients have cutaneous involvement, and skin lesions are often the first manifestation that typically result in medical attention (14, 15). The skin lesions are usually manifested as bruise-like lesions, plaques or nodules on the head, trunk, and extremities (6). A small percentage of patients will present with isolated skin disease, while the majority of patients has extracutaneous manifestations, including lymphadenopathy, fever, fatigue, or testicular swelling, but rarely have massive splenomegaly or hypersplenism (15–19).

The diagnosis of BPDCN is mainly based on histology, immunohistochemistry, and flow cytometry (3, 4, 20). Under microscope, the tumor cells are medium sized with a slightly irregular-shaped nucleus and smooth chromatin, similar to lymphoblasts (14). IHC usually demonstrates that the characteristic markers of plasmacyte-like dendritic cells, such as CD4, CD56, CD123, CD303 and T-cell leukemia/lymphoma protein 1 (TCL-1), are positive, while the markers of myelocyte, T/B lymphocyte, and NK cell are negative (3, 4, 21).

Currently, only a few case reports have described the imaging features of BPDCN on ^18^F-FDG PET/CT. Typical presentations include mildly hypermetabolic cutaneous lesions with thickened skin or subcutaneous mass, mildly hypermetabolic enlarged lymph nodes in multiple regions, and slightly higher ^18^F-FDG uptake in bone marrow, with reported SUVmax range of 1.9–2.4 (17, 22–24). Although the imaging features of BPDCN on ^18^F-FDG PET/CT are nonspecific, ^18^F-FDG PET/CT is crucial in staging of BPDCN by detecting the involvement of regional lymph node, bone marrow, and other organs, which is related to prognosis of the disease (5, 17).

BPDCN is an aggressive hematopoietic malignancy and exhibits a poor prognosis, with median survival of less than 2 years (25). There is no standard treatment-protocol for BPDCN, and the commonly used chemotherapy regimens derive from other more common hematological malignancies, such as non-Hodgkin lymphoma (NHL), acute lymphoblastic leukemia (ALL) and acute myeloid leukemia (AML) (6, 8, 26). Patients with BPDCN can achieve remission after receiving leukemia-type chemotherapy, but the disease usually tends to relapse after a few months. Consequently, allogeneic HSCT is considered as a consolidative strategy to maintain sustained complete remission (20). In addition, in view of the highly aggressive clinical behavior of BPDCN, it is recommended to start HSCT as soon as possible (9, 22).

PTLD is a group of conditions involving abnormal lymphoid cells proliferation in the context of extrinsic immunosuppression after SOT or HSCT. It is one of the most serious complications after the transplantation (10). The incidence of PTLD is 1 to 20% of recipients overall and varies by transplanted organs. Recipients of intestine and multi-organ transplantation have the highest risk (12%–17%), followed by lung (6%–10%), heart (3%–5%), liver (2%–3%) and kidney (1.5%–2.5%), while the recipients of HSCT have the lowest risk (< 2%) (27, 28). Although the incidence of PTLD in HSCT recipients is relatively low, patients with the following risk factors are more prone to develop PTLD: the use of antithymocyte globulin or alemtuzumab, *in-vivo* T-cell depletion, EBV serology donor/recipient mismatch (recipient-negative/donor-positive), human leukocyte antigen (HLA) mismatch, splenectomy, a second HSCT, and so on (29).

Compared with the SOT-associated PTLDs, which usually demonstrated a bimodal time distribution, with an early peak in the first 2 years and a second peak between 5 and 10 years after transplantation, most of the HSCT-associated PTLDs only show the early-onset pattern (10, 30). In many early-onset cases, carcinogenic EBV is a key pathogenic driver. The immunosuppressive therapy after allogeneic HSCT may disrupt the normal balance between the proliferation of potentially infected B-cells and the EBV-specific T-cell response, while the increased number of latently infected B cells may develop into PTLD (31). Furthermore, the splenectomy before HSCT may impair the function of CD5 positive B cell and lead to explosive growth of EBV load (32).

According to the World Health Organization (WHO), PTLDs was classified into four main types, namely, early lesions, polymorphic PTLD, monomorphic PTLD (with many subtypes), and classic Hodgkin lymphoma-like PTLD. EBV-related PTLD may evolve from a polyclonal disorder to a more aggressive monoclonal variant. Therefore, early diagnosis, accurate staging, and timely treatment are of vital importance to reduce morbidity and mortality. The European Conference in Infections in Leukemia recommended in the evidence-based guidelines that weekly EBV-DNA screening should be carried out on high-risk allogeneic HSCT recipients for at least 3 months (33).

The clinical manifestations of PTLD are usually nonspecific, and the patients commonly present with fever, anorexia, lethargy, night sweats, and weight loss. When latently-infected lymphocytes expand within the reticuloendothelial system, patients can present with lymphadenopathy, symptomatic hepatosplenomegaly, ascites, abdominal pain and cytopenias (34). Clinicians should differentiate PTLD from an acute infection, GVHD, graft rejection or malignancy relapse. Surveillance and diagnostic strategies for high-risk population of PTLD include routine blood test, weekly EBV DNA monitoring, imaging and tissue biopsy. Pathological diagnosis is the gold standard of PTLD, and most importantly, it can determine the PTLD subtype, which forms the basis for the subsequent treatment planning.

^18^F-FDG PET/CT may provide additional evidence for supporting or refuting the suspicion of PTLD, and suggest lesions accessible for biopsy (21, 35–37). As ^18^F-FDG PET/CT can provide both anatomic and metabolic information of lesions, it can increase the sensitivity of detecting occult metabolically active lesions or small lesions neglected on CT scan due to not meeting size criteria (35). Besides, it was reported that ^18^F-FDG PET/CT had advantage in detecting extra-nodal lesions due to its additional metabolic information (38). A recent retrospective study including 91 patients with clinical suspicion of PTLD showed that the sensitivity and specificity of ^18^F-FDG PET/CT for the detection of PTLD were 85 and 90%, respectively (30). Another monocentric retrospective analysis involving 170 cases showed that ^18^F-FDG uptake in PTLD was generally high with a median SUVmax of 17.4 (range 2.6–26.4) (39). However, ^18^F-FDG uptake will also increase in other conditions in patients after transplantation, such as postsurgical inflammation, infection, bone marrow activation and transplant rejection. In these cases, the detection of non-specific ^18^F-FDG-avid lesions will make the diagnosis of PTLD challenging.

It is worth noting that in this case, there were small foci of slightly increased uptake in the bone marrow (heterogenous uptake), slightly increased uptake in lymph nodes and skin in BPDCN, whereas there was intense uptake in evidently enlarged lymph nodes in PTLD, consistent with previous reports (17, 22–24, 39). This indicates that the differences in ^18^F-FDG uptake patterns may also be a distinguishing point between these two diseases.

As a whole-body functional and molecular imaging method, ^18^F-FDG PET/CT has unique advantages in staging and re-staging. Accurate staging is required for clinicians to decide the management strategy, as well as to evaluate the treatment response promptly and accurately. At present, the ^18^F-FDG PET/CT staging procedures of lymphoma are adopted for PTLD staging in many centers (40).

According to the National Comprehensive Cancer Network (NCCN) guideline (41), the main treatment options for PTLD include reducing immunosuppression, rituximab monotherapy, chemotherapy with or without rituximab, sequential chemo-immunotherapy, and so on.

In summary, we herein report a rare case of a patient with BPDCN who suffered from PTLD after allogeneic HSCT. This case indicates when new enlarged lymph nodes after HSCT and immunosuppressive therapy in patients with BPDCN are discovered, PTLD should be taken into consideration. ^18^F-FDG PET/CT examination may provide additional evidence for supporting or refuting the suspicion of PTLD, and suggest lesions accessible for biopsy.

## Data availability statement

The original contributions presented in the study are included in the article/supplementary material, further inquiries can be directed to the corresponding authors.

## Ethics statement

Written informed consent was obtained from the individual(s) for the publication of any potentially identifiable images or data included in this article.

## Author contributions

JC and XZ: imaging data analysis, manuscript draft, and editing. LM and YG: imaging data collection. ZF and ML: supervision, writing review, and editing. All authors contributed to the article and approved the submitted version.

## Funding

The author(s) declare that no financial support was received for the research, authorship, and/or publication of this article.

## Conflict of interest

The authors declare that the research was conducted in the absence of any commercial or financial relationships that could be construed as a potential conflict of interest.

## Publisher’s note

All claims expressed in this article are solely those of the authors and do not necessarily represent those of their affiliated organizations, or those of the publisher, the editors and the reviewers. Any product that may be evaluated in this article, or claim that may be made by its manufacturer, is not guaranteed or endorsed by the publisher.
